# Supplementary material for: Role of the primate ventral striatum as a neural hub bridging option valuation and action selection
Source: Nat Commun. 2026 Mar 28;17:2501. doi: 10.1038/s41467-026-70634-6 (PMC13032925; doi:10.1038/s41467-026-70634-6)
Supplement: Supplementary file 1 — Supplementary Information [file 41467_2026_70634_MOESM1_ESM.pdf]

Supplementary Information to:

Role of the primate ventral striatum as a neural hub bridging option valuation and action selection

Masafumi Nejime[1], Mengxi Yun[1], Yawei Wang[1], Takashi Kawai[1], Jun Kunimatsu[1,2], Hiroshi Yamada[1,2], Ken-ichi Inoue[3], Masahiko Takada[3], Masayuki Matsumoto[1,2,3,*]

[1] Institute of Medicine, University of Tsukuba, Tsukuba, Ibaraki 305-8575, Japan

[2] Transborder Medical Research Center, University of Tsukuba, Tsukuba, Ibaraki 305-8575, Japan

[3] Center for the Evolutionary Origins of Human Behavior, Kyoto University, Inuyama, Aichi 484-8506, Japan

[*] Correspondence to: Masayuki Matsumoto (matsumoto.masayuki.4w@kyoto-u.ac.jp)

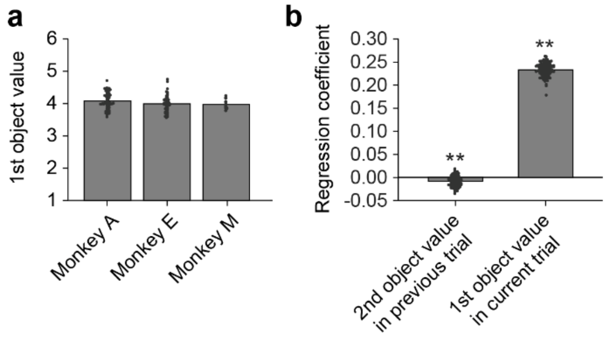

Supplementary Figure 1. **Risk attitude and effect of recent experience on action selection. a** Risk attitudes of monkey A (*n* = 71 sessions), monkey E (*n* = 54 sessions) and monkey M (*n* = 18 sessions) quantified as the value of the first object that was chosen with 50% probability. **b** Effects of the second object value in the previous trial and the first object value in the current trial on the monkey's action selection (*n* = 143 sessions). Double asterisks indicate a significant difference from zero (second object value in previous trial: $p = 8.4 \times 10^{-13}$; first object value in current trial: $p = 3.2 \times 10^{-25}$; two-tailed Wilcoxon signed-rank test). Small plots represent individual data points, and bar graphs indicate mean values.

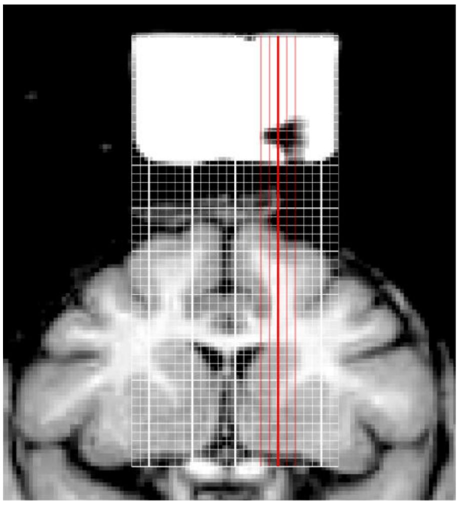

Supplementary Figure 2. **Penetrations of recording electrodes.** The penetrations of recording electrodes at a representative anterior-posterior position (A25) in the right ventral striatum of monkey E are shown as red lines on magnetic resonance imaging (MRI). The recording chamber is also visible in the MRI. Each vertical line represents a penetration hole in a recording grid, spaced at 1-mm intervals.

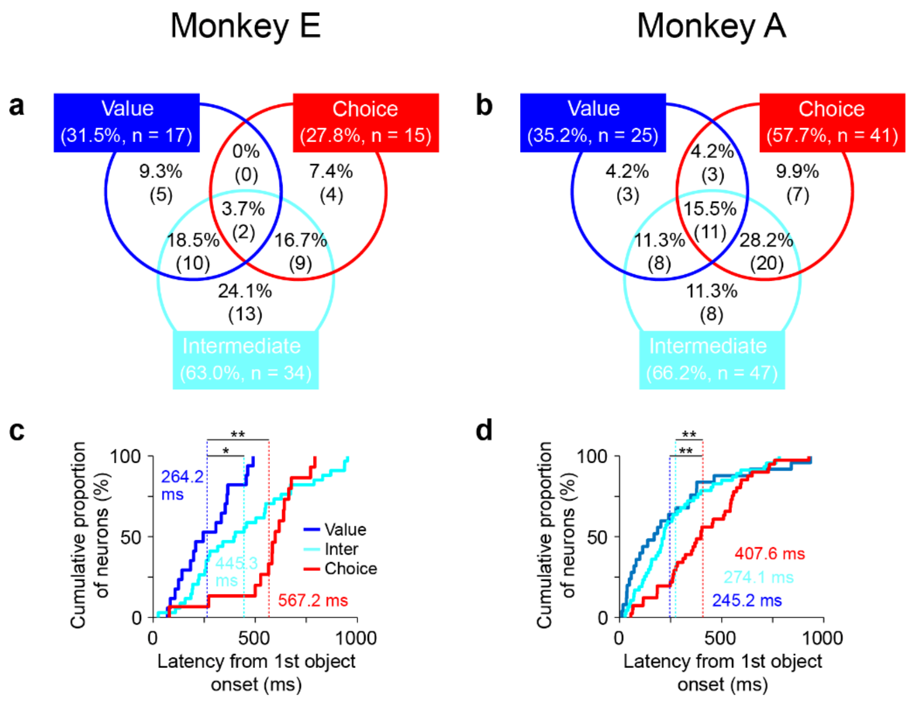

Supplementary Figure 3. **Neural signature of option valuation and action selection for individual monkeys. a**, **b** Proportion of neurons with value-modulated, intermediate, and choice-modulated signals for monkey E (**a**) and monkey A (**b**). Conventions follow those used in Fig. 2f. **c**, **d** Cumulative histograms of the latencies of the value-modulated, intermediate and choice-modulated signals aligned at the onset of the first object for monkey E (**c**) and monkey A (**d**). Conventions follow those used in Fig. 2g. Single and double asterisks indicate a significant difference between the latencies in (**c**) (value vs. intermediate: p = 0.019; value vs. choice: p= 1.0 × 10^{-4}; two-tailed Wilcoxon signed-rank test) and in (**d**) (value vs. choice: p = 1.6 × 10^{-3}; intermediate vs. choice: p = 2.0 × 10^{-3}; two-tailed Wilcoxon signed-rank test).

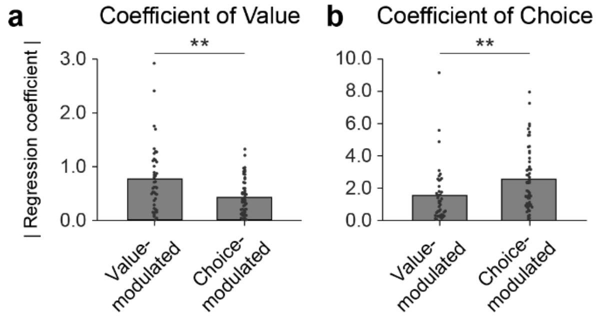

Supplementary Figure 4. **Effects of value and choice on value-modulated and choice-modulated signals determined by ridge regression.** Absolute value of regression coefficients for the first object value (**a**) and whether the monkey released the button to choose the object (**b**) are shown for neurons with value-modulated signal (*n* = 42) and choice-modulated signal (*n* = 56). Double asterisks denote a significant difference between the two groups in (**a**) ($p = 2.3 \times 10^{-3}$; two-tailed Wilcoxon rank-sum test) and in (**b**) ($p = 1.4 \times 10^{-3}$; two-tailed Wilcoxon rank-sum test). Error bars indicate SEM. Small plots represent individual data points, and bar graphs indicate mean values.

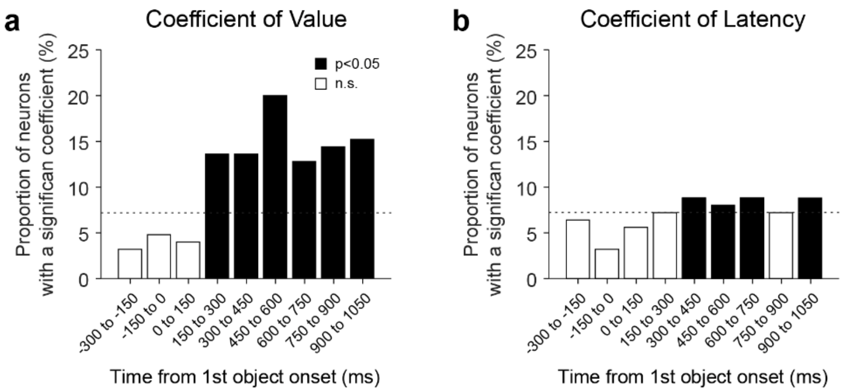

Supplementary Figure 5. **Effects of value and latency on neuronal activity determined by ridge regression.** Proportions of neurons with a significant regression coefficient for the first object value (**a**) and the latency of button release (**b**) are shown for each 150-ms time window after the first object onset (n = 125). Dotted line indicates the chance level calculated by a Monte Carlo analysis (p = 0.05; two-tailed Monte Carlo test), in which the multiple-comparison problem across time windows was controlled using a max-statistic permutation procedure. Filled bars indicate time windows in which the observed proportion exceeded the chance level.

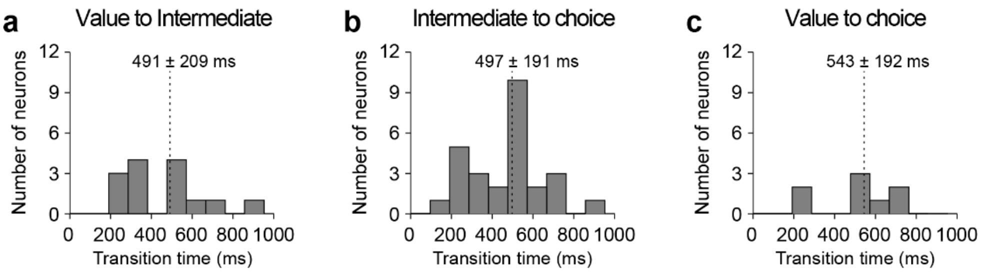

Supplementary Figure 6. **Time at which value-to-intermediate, intermediate-to-choice, and value-to-choice transitions occurred.** Distributions of the latencies of the later signals are shown for value-to-choice transition (*n* = 14) (**a**), intermediate-to-choice transition (*n* = 27) (**b**), and value-to-choice transition (*n* = 8) (**c**). Dotted line in each panel indicates the mean latency of the respective signals.

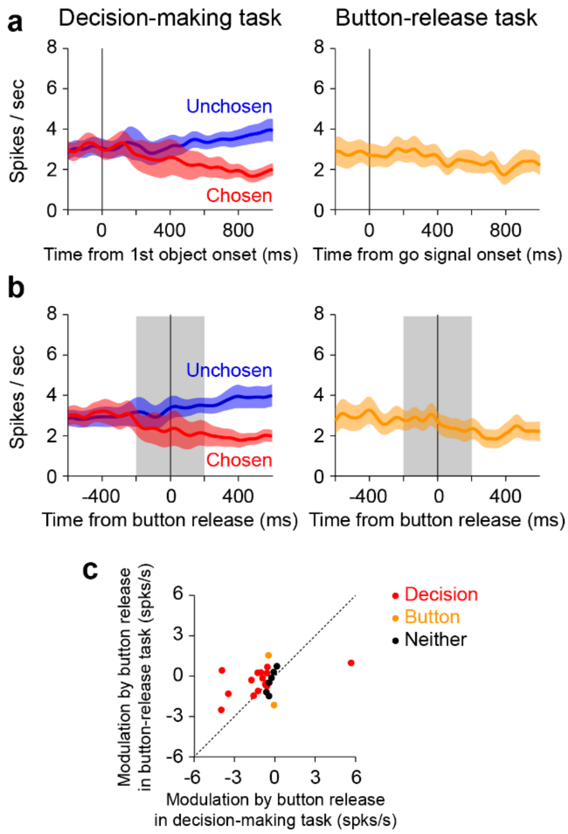

Supplementary Figure 7. **Negative neuronal modulation evoked by simple motor action. a**, **b** Averaged activity of 23 neurons representing choice-modulated signal with a negative modulation aligned at the first object onset (**a**) and the button release onset (**b**) in the decision-making task (left) and the button-release task (right). Color shaded areas indicate SEM. Conventions follow those used in Fig. 3b and 3c. **c** Comparison of the negative modulation magnitude evoked by button release between the decision-making task (x-axis) and the button-release task (y-axis) during the gray shading epoch in (**b**). Conventions follow those used in Fig. 3d.

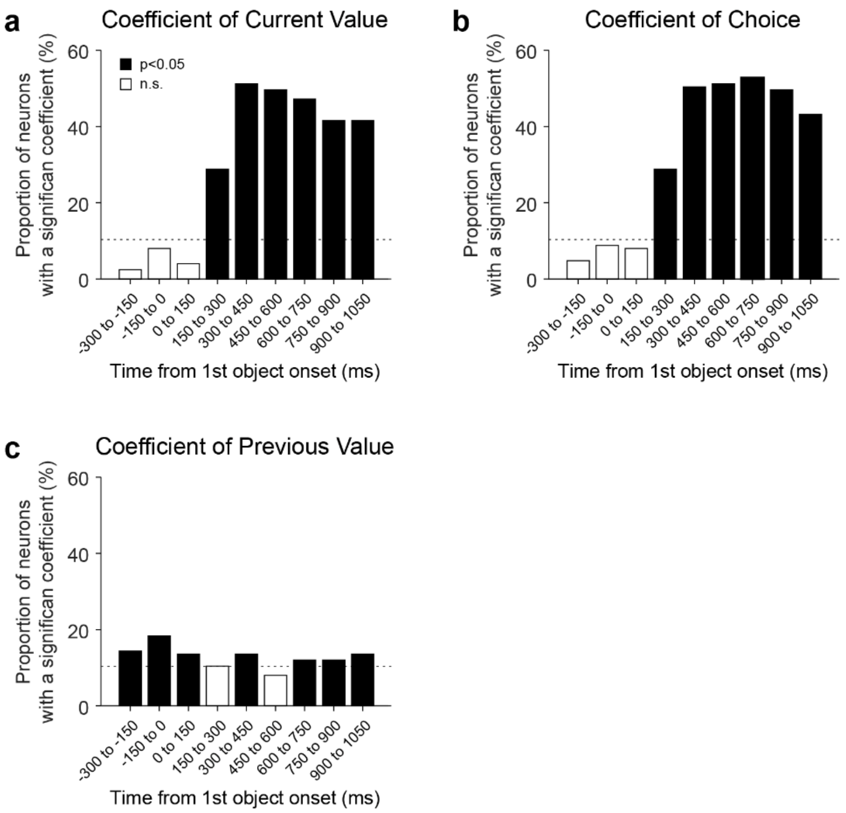

Supplementary Figure 8. **Effects of current value, choice, and previous value on neuronal activity determined by ridge regression.** Proportions of neurons with a significant regression coefficient for the first object value in the current trial (**a**), whether the monkey released the button to choose the first object (**b**), and the second object value in the previous trial (**c**) are shown (n = 125). Conventions follow those used in Supplementary Fig. 5.

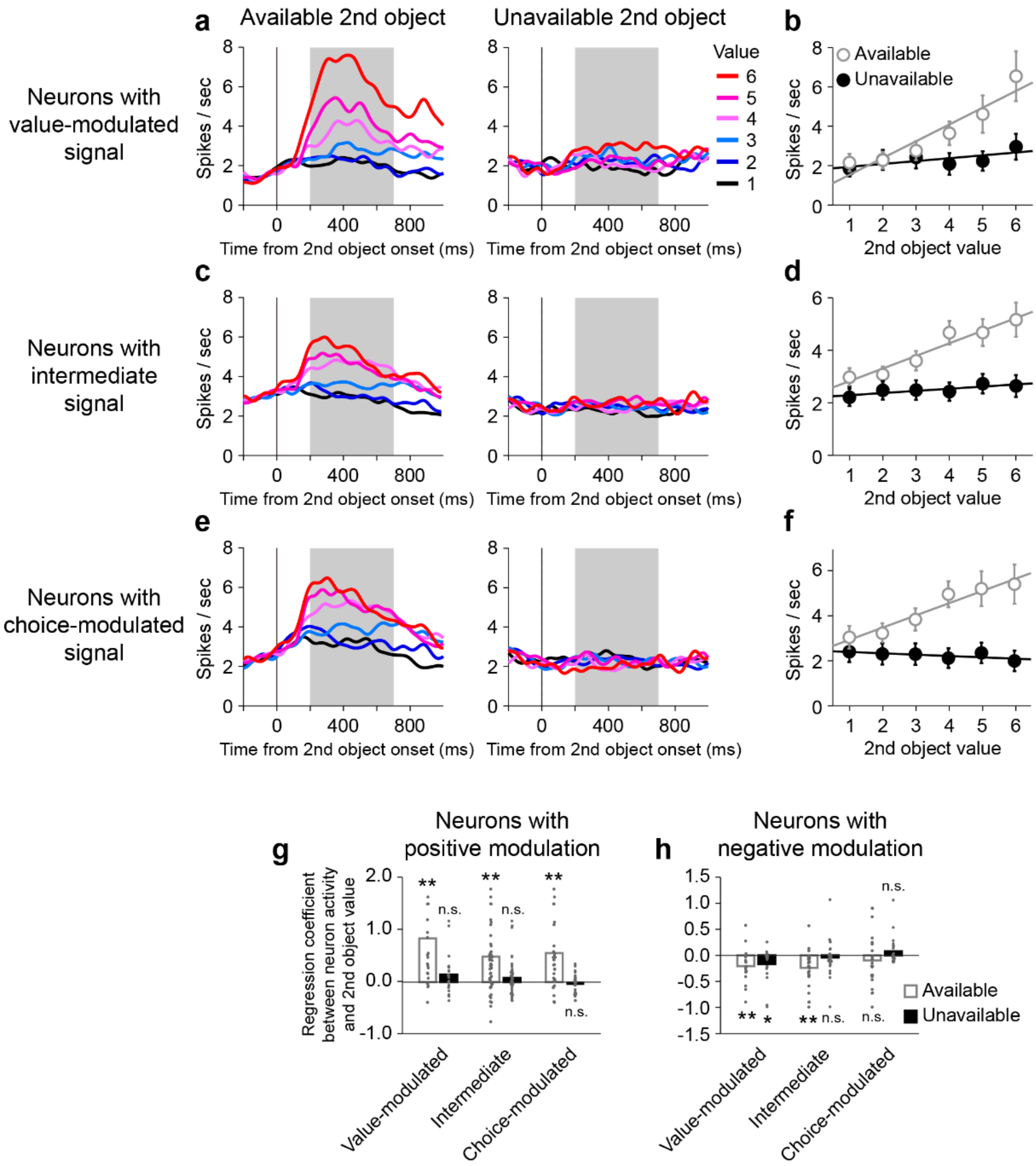

Supplementary Figure 9. **Modulations of neurons with value-modulated, intermediate, and choice-modulated signals evoked by the second object. a**, **c**, **e** Averaged activities of 23 neurons representing value-modulated signal (**a**), 51 neurons representing intermediate signal (**c**), and 33 neurons representing choice-modulated signal (**e**) with a positive modulation aligned at the second object onset. Spike density functions (SDFs) are shown for trials in which the monkey did not choose the first object (left, available 2nd object) and trials in which the monkey chose the first object

(right, unavailable 2nd object). Gray shaded areas indicate the time window to calculate the magnitude of neuronal activity. **b**, **d**, **f** Magnitude of activity plotted against the second object value shown for neurons representing value-modulated signal (**b**), intermediate signal (**d**), and choice-modulated signal (**f**) with a positive modulation. The magnitude is shown for available second object trials (open gray circles) and unavailable second object trials (filled black circles). Error bars indicate SEM. **g**, **h** Regression coefficient between neuronal activity and the second object value shown for each neuron type with a positive modulation (**g**) and with a negative modulation (**h**). The coefficient is shown for available second object trials (open gray bars) and unavailable second object trials (filled black bars). Double/single asterisks and n.s. denote significant and non-significant differences, respectively, from zero in (**g**) (value-modulated neurons for available: $p = 1.6\times 10^{-3}$ and for unavailable: $p = 0.09$; intermediate neurons for available: $p = 9.3 \times 10^{-5}$ and for unavailable: $p = 0.24$; choice-modulated neurons for available: $p = 2.3 \times 10^{-3}$ and for unavailable: $p = 0.15$; two-tailed Wilcoxon signed-rank test) and in (**h**) (value-modulated neurons for available: $p = 8.9 \times 10^{-3}$ and for unavailable: $p = 0.024$; intermediate neurons for available: $p = 1.4 \times 10^{-4}$ and for unavailable: $p = 0.10$; choice-modulated neurons for available: $p = 0.25$ and for unavailable: $p = 0.63$; two-tailed Wilcoxon signed-rank test). Small plots represent individual data points, and bar graphs indicate mean values.

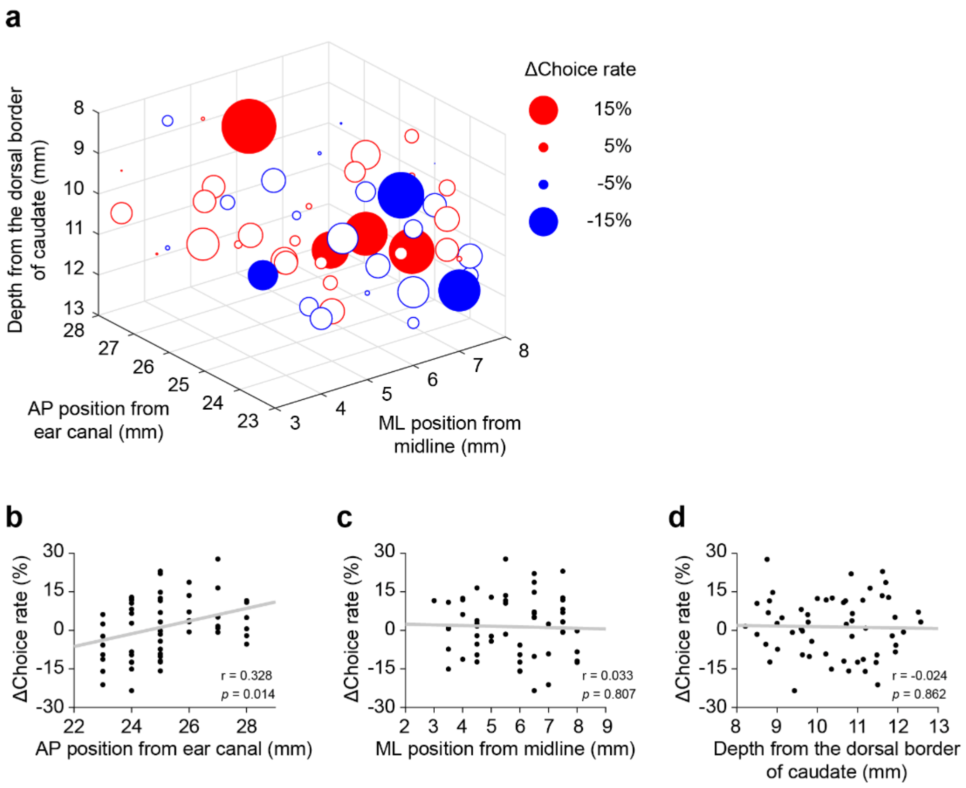

Supplementary Figure 10. **Locations of electrical stimulations affecting the choice rate of the first object with value 4. a** 3D map showing the effects of electrical stimulation on choice rate of the first object with value 4 (n = 56 sites). Red and blue circles indicate positive and negative changes in the choice rate (Δchoice rates), respectively. The size of each circle represents the magnitude of Δchoice rate. Filled circles indicate Δchoice rates that are significantly positive or negative compared to zero ($p < 0.05$; two-tailed chi-square test). **b-d** Correlations between Δchoice rate and anterior-posterior position (**b**), medial-lateral position (**c**), and depth from the dorsal border of the caudate (**d**). Grey lines indicate the regression lines.

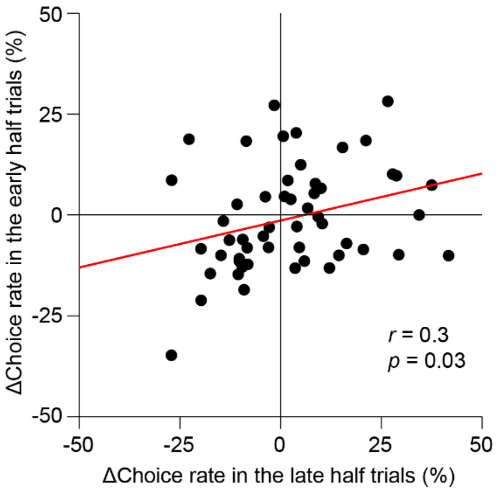

Supplementary Figure 11. **Comparison of the effect of electrical stimulation on the choice rate of the first object with value 4 between the early and late halves of the trials for each stimulation site.** ΔChoice rate (i.e., the difference in choice rate between stimulation and non-stimulation trials) for the first object with value 4 is plotted for the early half of the trials (y-axis) against the late half (x-axis) across stimulation sites. Red line indicates the regression line. Data for this analysis were obtained from 53 of the 56 stimulation sites that included at least 30 stimulation trials with the first object of value 4. These Δchoice rates exhibited a significantly positive correlation (r = 0.3, F = 4.9, p = 0.03; Pearson’s correlation test).

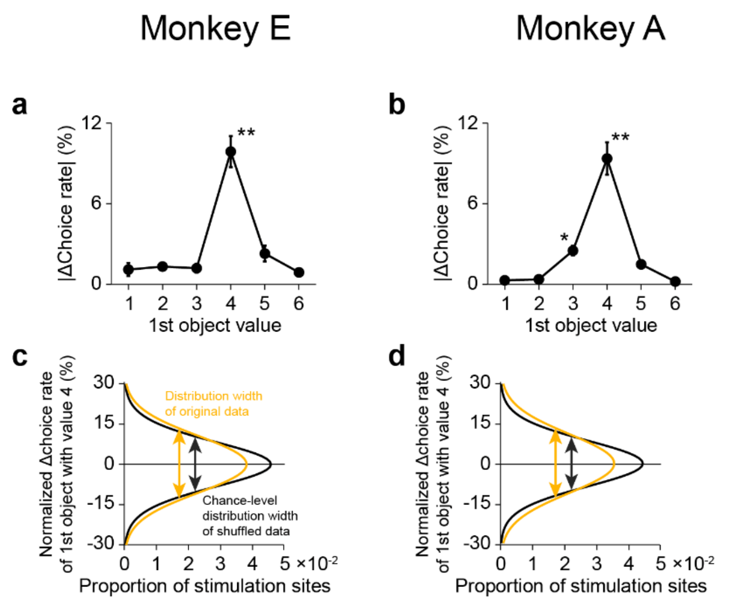

Supplementary Figure 12. **Effect of electrical stimulation of the ventral striatum for individual monkeys. a**, **b** Mean absolute Δchoice rate for each first object value for monkey E (n = 20 sites) (**a**) and monkey A (n = 36 sites) (**b**). Conventions follow those used in Fig. 4f. Double and single asterisks denote a significant difference in (**a**) (p = 5.3×10^{-21}; one-way ANOVA and post-hoc test) and in (**b**) (p = 4.7×10^{-31}; one-way ANOVA and post-hoc test). **c**, **d** Gaussian functions fitted to the Δchoice rate distribution of the original data (yellow curve) and the chance-level distribution of shuffled data (black curve) for monkey E (**c**) and monkey A (**d**). Conventions follow those used in Fig. 4g. The distribution width of the choice rate difference was significantly larger than the chance-level distribution (monkey E: p = 0.004; monkey A: p = 0.004; two-tailed Monte Carlo test).

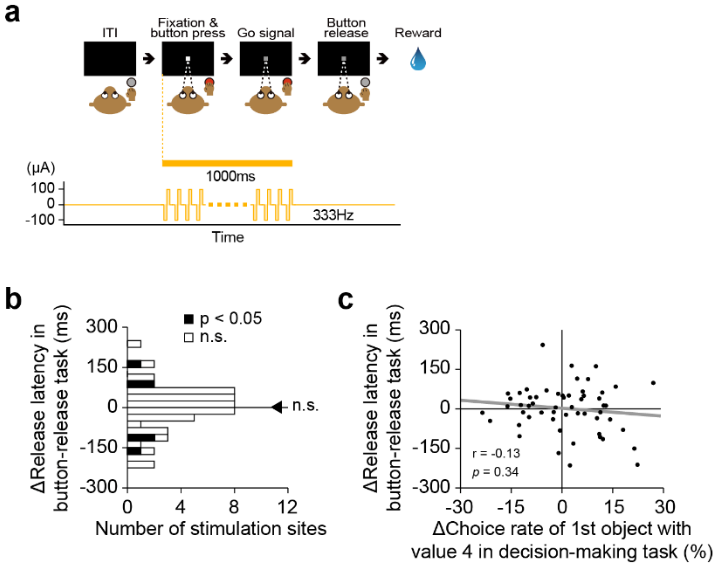

Supplementary Figure 13. **Effect of electrical stimulation of the ventral striatum on button-release latency in the button-release task. a** Electrical stimulation period during the button-release task. ITI, intertrial interval. **b** Distribution of the differences in button-release latency between stimulation and non-stimulation trials (Δrelease latency) (n = 56 sites). Filled bars represent Δrelease latency that are significantly different from zero (p < 0.05; two-tailed Wilcoxon signed-rank test). Black arrow indicates the mean Δrelease latency. n.s. denotes no significance (p = 0.65; two-tailed Wilcoxon signed-rank test). **c** Comparison between the Δchoice rates of the first object with value 4 and Δrelease latency (n = 56 sites). Each dot represents each stimulation site. Grey line indicates the regression line. (**a**) is based on [Yun et al., Science Advances, 2020 and 2023] and is reused under a CC BY license.

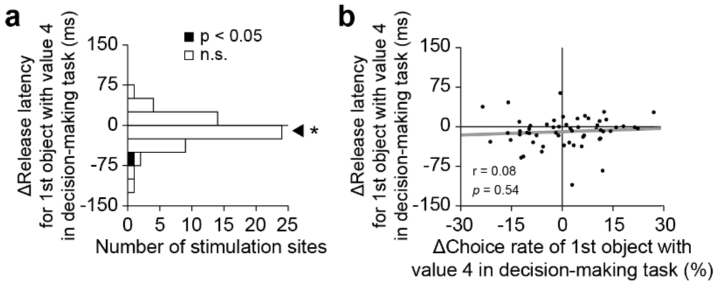

Supplementary Figure 14. **Effect of electrical stimulation of the ventral striatum on button-release latency in the decision-making task.** Conventions follow those used in Supplementary Fig. 13b and 13c. The analyses were performed on the same samples used in Supplementary Fig. 13b and 13c (n = 56 sites). Single asterisk denotes a significant difference from zero in (**a**) (p = 0.014; two-tailed Wilcoxon signed-rank test).

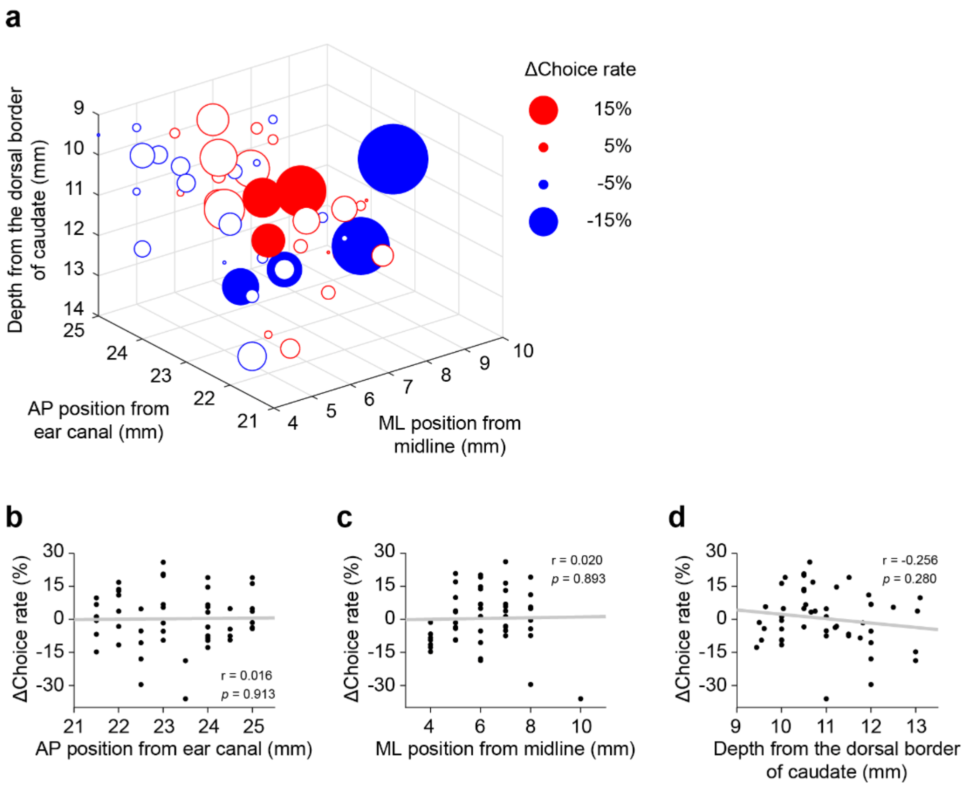

Supplementary Figure 15. **Locations of optogenetic facilitation affecting the choice rate of the first object with value 4.** Conventions follow those used in Supplementary Fig. 10. The analyses were performed using the samples obtained by the optogenetics experiment (*n* = 50 sites).

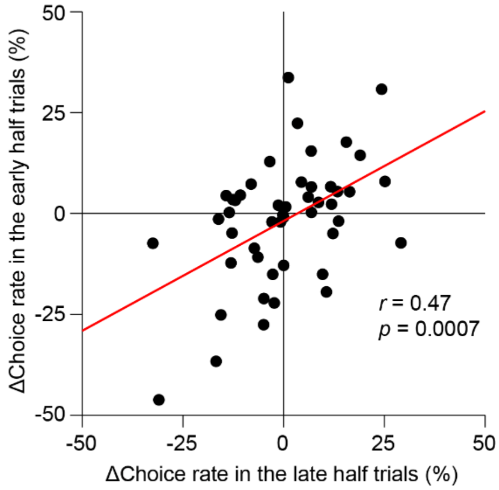

Supplementary Figure 16. **Comparison of the effect of optogenetic facilitation on the choice rate of the first object with value 4 between the early and late halves of the trials for each stimulation site.** Conventions follow those used in Supplementary Fig. 11. Data for this analysis were obtained from 48 of the 50 stimulation sites that included at least 30 stimulation trials with the first object of value 4. There was a significantly positive correlation (r = 0.47, F = 13.3, p = 0.0007; Pearson's correlation test).

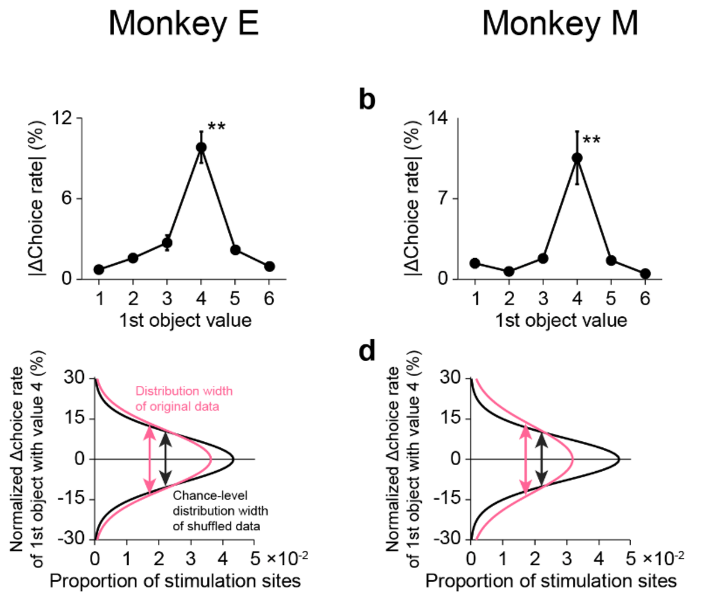

Supplementary Figure 17. **Effect of optogenetic facilitation of dopamine input to the ventral striatum for individual monkeys. a**, **b** Mean absolute Δchoice rate for each first object value for monkey E (*n* = 32 sites) (**a**) and monkey M (*n* = 18 sites) (**b**). Conventions follow those used in Fig. 5i. Double asterisks denote a significant difference in (**a**) ($p = 4.2 \times 10^{-26}$; one-way ANOVA and post-hoc test) and in (**b**) ($p = 1.9 \times 10^{-11}$; one-way ANOVA and post-hoc test). **c**, **d** Gaussian functions fitted to the Δchoice rate distribution of the original data (pink curve) and the chance-level distribution of shuffled data (black curve) for monkey E (**c**) and monkey M (**d**). Conventions follow those used in Fig. 5j. The distribution width of the choice rate difference was significantly larger than the chance-level distribution (monkey E: p = 0.002; monkey A: p = 0.002; two-tailed Monte Carlo test).

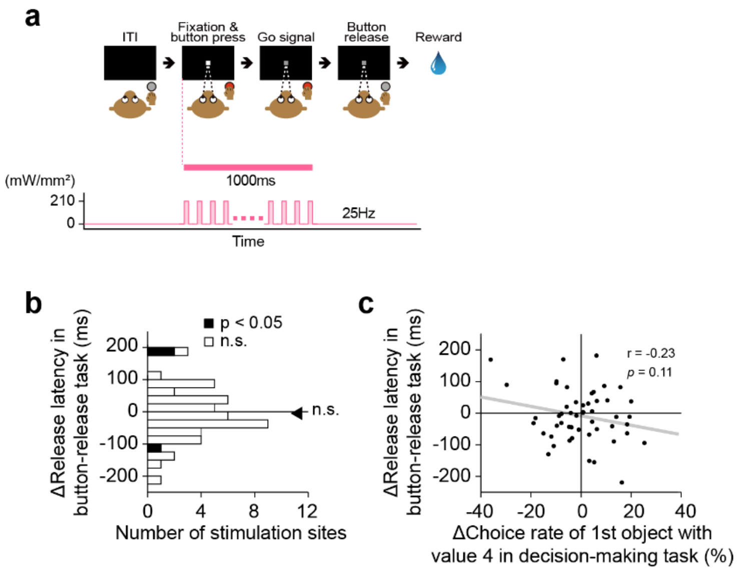

Supplementary Figure 18. **Effect of optogenetic facilitation of dopamine input to the ventral striatum on button-release latency in the button-release task.** Conventions follow those used in Supplementary Fig. 13. The analyses were performed using the samples obtained by the optogenetics experiment (n = 50 sites). n.s. denotes a non-significant difference from zero in (**b**) (p = 0.57; two-tailed Wilcoxon signed-rank test). (**a**) is based on [Yun et al., Science Advances, 2020 and 2023] and is reused under a CC BY license.

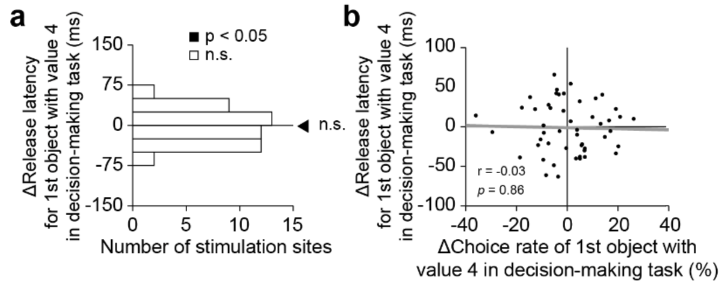

Supplementary Figure 19. **Effect of optogenetic facilitation of dopamine input to the ventral striatum on button-release latency in the decision-making task.** Conventions follow those used in Supplementary Fig. 13b and 13c. The analyses were performed on the same samples used in Supplementary Fig. 18b and 18c (n = 50 sites). n.s. denotes a non-significant difference from zero in (**a**) (p = 0.57; two-tailed Wilcoxon signed-rank test).
