# Supplementary material for: Role of the primate ventral striatum as a neural hub bridging option valuation and action selection
Source: Nat Commun. 2026 Mar 28;17:2501. doi: 10.1038/s41467-026-70634-6 (PMC13032925; doi:10.1038/s41467-026-70634-6)
Supplement: Supplementary file 2 — Reporting Summary [file 41467_2026_70634_MOESM2_ESM.pdf]

nature portfolio

Corresponding author(s): Masayuki Matsumoto

Last updated by author(s): Jan 27, 2026

# Reporting Summary

Nature Portfolio wishes to improve the reproducibility of the work that we publish. This form provides structure for consistency and transparency in reporting. For further information on Nature Portfolio policies, see our Editorial Policies and the Editorial Policy Checklist.

## Statistics

For all statistical analyses, confirm that the following items are present in the figure legend, table legend, main text, or Methods section.

| n/a | Confirmed | |
|---|---|---|
| ☐ | ☒ | The exact sample size (*n*) for each experimental group/condition, given as a discrete number and unit of measurement |
| ☐ | ☒ | A statement on whether measurements were taken from distinct samples or whether the same sample was measured repeatedly |
| ☐ | ☒ | The statistical test(s) used AND whether they are one- or two-sided<br>*Only common tests should be described solely by name; describe more complex techniques in the Methods section.* |
| ☐ | ☒ | A description of all covariates tested |
| ☐ | ☒ | A description of any assumptions or corrections, such as tests of normality and adjustment for multiple comparisons |
| ☐ | ☒ | A full description of the statistical parameters including central tendency (e.g. means) or other basic estimates (e.g. regression coefficient) AND variation (e.g. standard deviation) or associated estimates of uncertainty (e.g. confidence intervals) |
| ☐ | ☒ | For null hypothesis testing, the test statistic (e.g. *F*, *t*, *r*) with confidence intervals, effect sizes, degrees of freedom and *P* value noted<br>*Give P values as exact values whenever suitable.* |
| ☒ | ☐ | For Bayesian analysis, information on the choice of priors and Markov chain Monte Carlo settings |
| ☒ | ☐ | For hierarchical and complex designs, identification of the appropriate level for tests and full reporting of outcomes |
| ☐ | ☒ | Estimates of effect sizes (e.g. Cohen's *d*, Pearson's *r*), indicating how they were calculated |

*Our web collection on statistics for biologists contains articles on many of the points above.*

## Software and code

Policy information about availability of computer code

| | |
|---|---|
| Data collection | TEMPO system (Reflective Computing) for data collection and ASD (Apha omega) for online spike sorting. |
| Data analysis | Matlab R2020b (MathWorks) for all offline analyses. |

For manuscripts utilizing custom algorithms or software that are central to the research but not yet described in published literature, software must be made available to editors and reviewers. We strongly encourage code deposition in a community repository (e.g. GitHub). See the Nature Portfolio guidelines for submitting code & software for further information.

## Data

Policy information about availability of data

All manuscripts must include a data availability statement. This statement should provide the following information, where applicable:

- Accession codes, unique identifiers, or web links for publicly available datasets
- A description of any restrictions on data availability
- For clinical datasets or third party data, please ensure that the statement adheres to our policy

DATA AVAILABILITY: Source data are provided with this paper.
CODE AVAILABILITY: No custom codes or algorithms were developed for this study. All analyses were performed using standard MATLAB (MathWorks) functions, and the analysis procedures are fully described in the 'Methods' section. All codes used in this study are available from the corresponding author upon request.

## Research involving human participants, their data, or biological material

Policy information about studies with human participants or human data. See also policy information about sex, gender (identity/presentation), and sexual orientation and race, ethnicity and racism.

| | |
|---|---|
| Reporting on sex and gender | N/A |
| Reporting on race, ethnicity, or other socially relevant groupings | N/A |
| Population characteristics | N/A |
| Recruitment | N/A |
| Ethics oversight | N/A |

Note that full information on the approval of the study protocol must also be provided in the manuscript.

# Field-specific reporting

Please select the one below that is the best fit for your research. If you are not sure, read the appropriate sections before making your selection.

[x] Life sciences [ ] Behavioural & social sciences [ ] Ecological, evolutionary & environmental sciences

For a reference copy of the document with all sections, see nature.com/documents/nr-reporting-summary-flat.pdf

# Life sciences study design

All studies must disclose on these points even when the disclosure is negative.

| | |
|---|---|
| Sample size | We did not use any statistical method to determine sample sizes a priori. The sample sizes of animals (two monkeys for neurophysiology, two monkeys for electrical stimulation, and two monkeys for optogenetics), recorded neurons (125), electrical stimulation sites (56), and optogenetic stimulation sites (50) in the present study were comparable to those of previous studies in the field of systems neuroscience in nonhuman primates. |
| Data exclusions | We excluded 3 of the 56 electrical stimulation data and 2 of the 50 optogenetic stimulation data from the analysis to test the stability of the effects of the stimulations because these data did not include enough trials for this test (< 30 trials). These analyses are shown in Supplementary Fig. 11 and 16. |
| Replication | The main results were reliably reproduced in both monkeys. The individual monkeys' data are shown in Supplementary Fig. 3, 12, and 17. |
| Randomization | In the decision-making task, two of the 6 visual objects were randomly selected with replacement and presented as the first and second objects. In order to analyze neuronal activity, we used Monte Carlo procedures in which random resampling and comparison were repeated 1000 times. |
| Blinding | The investigators were not blind to the conditions of the experiments for data collection or analyses. However we did not select the type of neurons that we recorded during single-unit recording sessions, or behavioral effects in each stimulation sites . |

# Reporting for specific materials, systems and methods

We require information from authors about some types of materials, experimental systems and methods used in many studies. Here, indicate whether each material, system or method listed is relevant to your study. If you are not sure if a list item applies to your research, read the appropriate section before selecting a response.

### Materials & experimental systems

| n/a | Involved in the study | |
|---|---|---|
| [ ] | [x] | Antibodies |
| [x] | [ ] | Eukaryotic cell lines |
| [x] | [ ] | Palaeontology and archaeology |
| [ ] | [x] | Animals and other organisms |
| [x] | [ ] | Clinical data |
| [x] | [ ] | Dual use research of concern |
| [x] | [ ] | Plants |

### Methods

| n/a | Involved in the study | |
|---|---|---|
| [x] | [ ] | ChIP-seq |
| [x] | [ ] | Flow cytometry |
| [x] | [ ] | MRI-based neuroimaging |

# Antibodies

| | |
|---|---|
| Antibodies used | mouse anti-HA.11 clone 16B12 monoclonal IgG (901513, BioLegend, San Diego, USA)<br>donkey anti-mouse IgG biotin-SP-conjugated antibody (715-065-150, Jackson ImmunoResearch Laboratories, West Grove, USA)<br>donkey anti-mouse IgG conjugated with Alexa Fluor 488 (A21202, Jackson ImmunoResearch Laboratories, West Grove, USA)<br>donkey anti-rabbit IgG conjugated with Alexa Fluor 555 (A31572, Invitrogen, Waltham, USA)<br>donkey anti-guinea pig IgG conjugated with Alexa Fluor 647 (706-605-148, Jackson ImmunoResearch Laboratories, West Grove, USA) |
| Validation | mouse anti-HA.11 clone 16B12 monoclonal IgG (901513, BioLegend, San Diego, USA): https://www.biolegend.com/ja-jp/products/anti-ha-11-epitope-tag-antibody-11071<br>donkey anti-mouse IgG biotin-SP-conjugated antibody (715-065-150, Jackson ImmunoResearch Laboratories, West Grove, USA): https://www.jacksonimmuno.com/catalog/products/715-065-150<br>donkey anti-mouse IgG conjugated with Alexa Fluor 488 (A21202, Jackson ImmunoResearch Laboratories, West Grove, USA): https://www.thermofisher.com/antibody/product/A-21202.html?CID=AFLCA-A-21202<br>donkey anti-rabbit IgG conjugated with Alexa Fluor 555 (A31572, Invitrogen, Waltham, USA): https://www.fishersci.com/shop/products/donkey-anti-rabbit-igg-h-l-highly-cross-adsorbed-secondary-antibody-alexa-fluor-555/A31572<br>donkey anti-guinea pig IgG conjugated with Alexa Fluor 647 (706-605-148, Jackson ImmunoResearch Laboratories, West Grove, USA): https://www.jacksonimmuno.com/catalog/products/706-605-148 |

# Animals and other research organisms

Policy information about studies involving animals; ARRIVE guidelines recommended for reporting animal research, and Sex and Gender in Research

| | |
|---|---|
| Laboratory animals | Three adult macaque monkeys (monkey E: Macaca mulatta, male, 11.7 kg, 15 years old; monkey A: Macaca mulatta, male, 8.4 kg, 11 years old; monkey M: Macaca fuscata, female, 6.3 kg, 6 years old) |
| Wild animals | N/A |
| Reporting on sex | Three adult macaques were used in this study (two males and one female). No systematic analysis of sex differences was performed because of the limited sample size. All experimental procedures were identical across animals regardless of sex. We have analyzed individual monkeys' data and found consistent trends between them, even between male and female (Supplementary Fig. 3, 12, and 17.) |
| Field-collected samples | N/A |
| Ethics oversight | All animal care and experimental procedures were approved by the Animal Experiment Committee and Genetic Modification Experiment Safety Committee in University of Tsukuba (permission number, 14-137) |

Note that full information on the approval of the study protocol must also be provided in the manuscript.

# Plants

| | |
|---|---|
| Seed stocks | N/A |
| Novel plant genotypes | N/A |
| Authentication | N/A |
